# Reverse Shoulder Megaprosthesis for Massive Proximal Humeral Bone Loss in Fracture Outcome Settings: A Report of Two Cases and Literature Review

**DOI:** 10.7759/cureus.54276

**Published:** 2024-02-15

**Authors:** Vincenzo Morea, Alberto Polizzi, Giuseppe Niccoli, Guido Zattoni, Luca Andriollo

**Affiliations:** 1 Orthopedics and Traumatology, Fondazione Poliambulanza Hospital, Brescia, ITA; 2 Department of Orthopedics, Università Cattolica del Sacro Cuore, Rome, ITA

**Keywords:** limb reconstruction, reverse proximal humerus replacement, navigation system, proximal humerus bone loss, reverse shoulder megaprosthesis

## Abstract

In trauma settings, including the management of outcomes, there is no consensus on the most appropriate reconstructive method in the presence of severe bone loss of the proximal humerus. The objective of this report is to evaluate the short-term functional outcomes of two patients in whom reverse shoulder megaprosthesis was used to treat the failure of trauma surgery with severe bone loss. The secondary objective was to compare the results obtained with the literature regarding the use of megaprosthesis in shoulder trauma surgery. The patients showed a satisfying functional recovery and increased quality of life. At the 12-month follow-up, no complications occurred. Regarding the risk of complications, especially the risks of mobilization of the megaprosthesis, the CT-based intraoperative navigation system optimizes the configuration of the screw for the initial fixation of the glenoid component. Shoulder megaprosthesis appears to be a viable option not only in oncologic surgery but also in cases of failed trauma surgery. The functional results, considering functional score and range of motion, are encouraging and allow patients to improve their quality of life.

## Introduction

The use of reverse shoulder arthroplasty has gradually increased over the past decade [1]. Indications have increased, not only for rotator cuff degeneration arthropathy but also for revision shoulder arthroplasty, complex proximal humerus fractures with rotator cuff deficiency, treatment of tumors of the proximal humerus, proximal humeral malunions or nonunions or inflammatory arthritis [2,3].

Reverse shoulder megaprosthesis is mainly used in oncological orthopedics, in cases of humerus bone resection. At midterm follow-up, the functional outcomes of patients who received a reverse shoulder megaprosthesis following orthopedic oncologic tumor resection in the proximal humerus were found to be satisfactory. Notable improvements were observed in the range of motion for elevation, abduction, and external rotation, as well as overall shoulder functioning [4].

The use of megaprosthesis is favored for several reasons, such as its ability to provide predictable functional outcomes, facilitate early rehabilitation after surgery, and offer intraoperative flexibility in the length of reconstruction, thanks to the modularity of the device. These advantages make megaprosthesis a preferred choice in treating certain conditions where functional outcome and postoperative recovery are crucial factors [5]. However, in traumatology, including the management of outcomes, there is no consensus on the most appropriate reconstructive method in the presence of severe bone loss of the proximal humerus.

This report describes two cases of short-term osteosynthesis failure treated using a modular reconstruction system and a literature review about the use of megaprosthesis in the treatment of proximal humerus complex fractures. The objective of this case report is to evaluate the mid- and short-term functional outcomes of two patients in whom reverse shoulder megaprosthesis was used to treat the failure of trauma surgery with severe bone loss. The secondary objective was to compare the results obtained with the literature regarding the use of megaprosthesis in shoulder trauma surgery.

## Case presentation

Case 1

**Figure 1:**
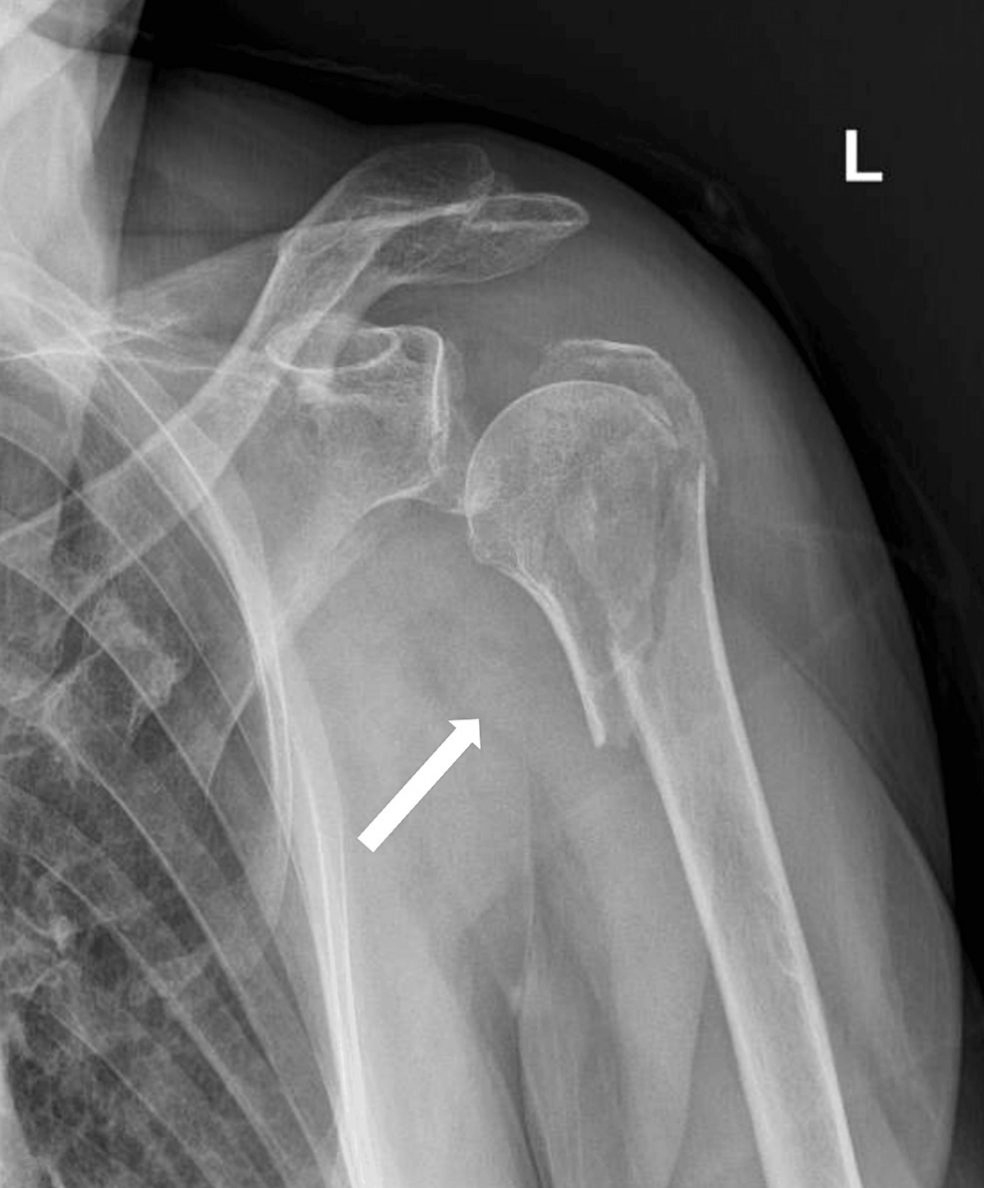
Multi-fragmented proximal meta-epiphyseal fracture of the humerus, as indicated by the arrow (Case 1) L: left

In September 2022, a 76-year-old female was admitted to the orthopedic emergency department with right shoulder pain following a domestic injury. The patient's medical history included only arterial hypertension. She led an active life, completely autonomously. Radiography revealed a multi-fragmented proximal meta-epiphyseal fracture of the humerus (AO/OTA 11C3) (Figure 1) and the patient did not present with significant local edema or soft tissue injury.

**Figure 2:**
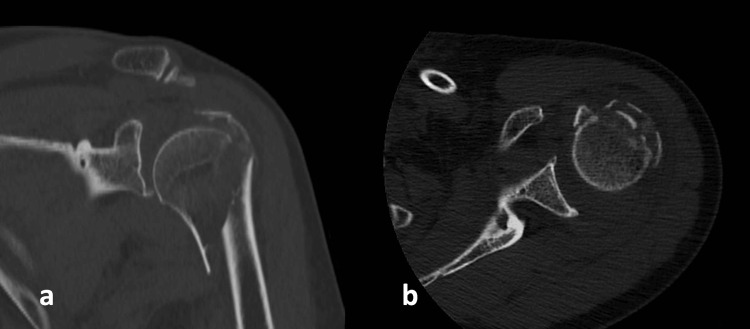
CT scan with coronal (a) and axial (b) views showing multi-fragmented proximal meta-epiphyseal fracture of the humerus (Case 1)

A CT scan was performed to further evaluate the extent of the fracture (Figure 2).

**Figure 3:**
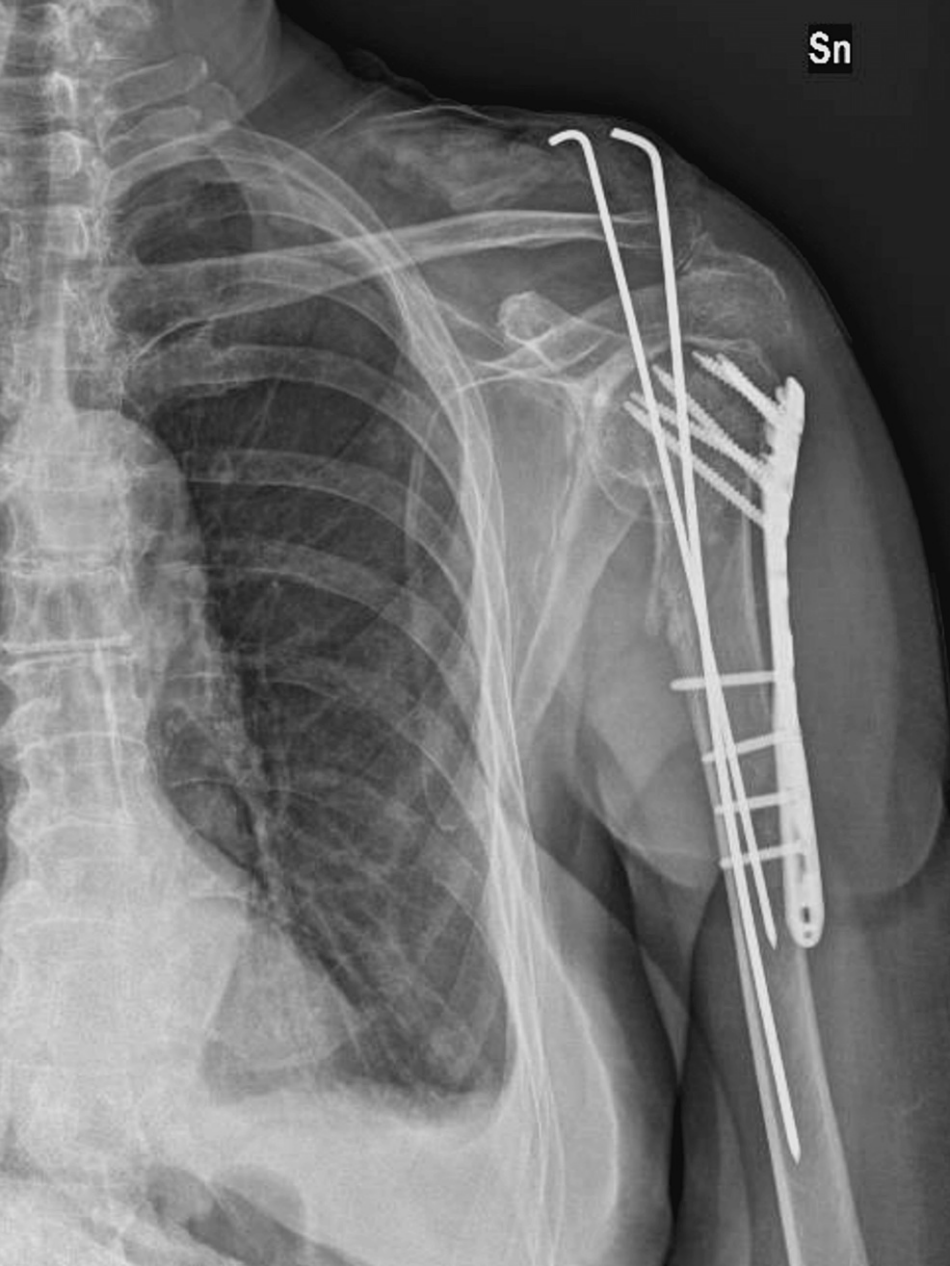
Post-surgical treatment open reduction internal fixation X-ray (Case 1) Sn: left

The patient underwent surgical intervention with open reduction and internal fixation using a plate, screws, and Kirschner wires (Figure 3). 

After nearly one month, the patient returned to the orthopedic emergency department with purulent drainage from the Kirschner wire sites and erythematous skin with positive thermal sensitivity. The C-reactive protein (CRP) level was significantly elevated (278.4 mg/L; cut-off < 5.0), indicating the possibility of an infection.

**Figure 4:**
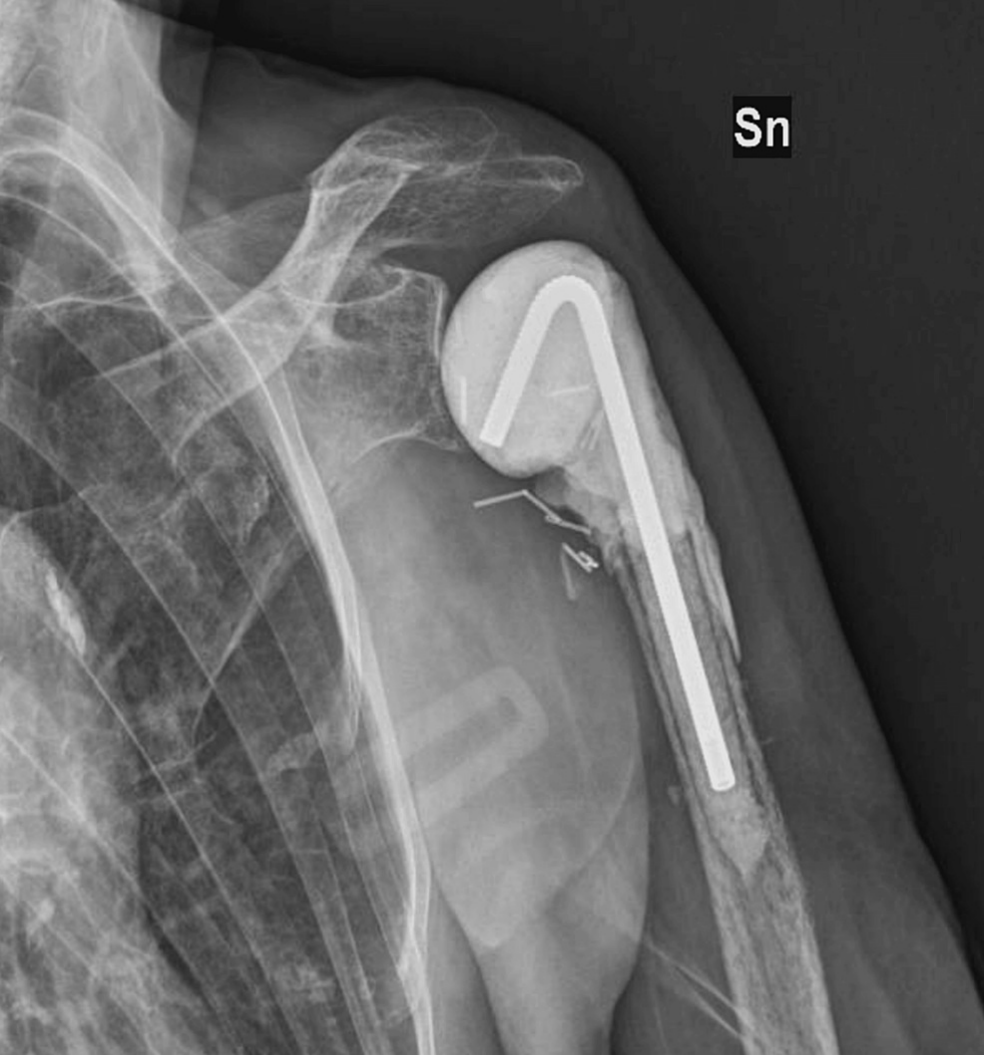
X-ray after implantation of antibiotic-loaded spacer (Case 1) Sn: left

In October 2022, the fixation devices were removed and a resection of the proximal humerus was performed. Plate and bone were sent for culture. Periarticular tissue leukocyte count revealed a value exceeding the cut-off of 5 granulocytes/5HPF. A rapid alpha-defensin Synovasure® (Zimmer Biomet Holdings, Inc., Warsaw, Indiana, United States) test was positive. Periarticular tissue samples were sent for culture, and an antibiotic-loaded spacer (TECRES S.p.A., Sommacampagna, Italy) was implanted (Figure 4).

Cultures revealed methicillin-sensitive *Staphylococcus aureus*. Upon consultation with an infectious disease specialist, the patient received sulfamethoxazole + trimethoprim for eight weeks. During a wash-out period, the patient's inflammatory indices were monitored. Additionally, clinical checks were performed to evaluate the surgical scar, which did not reveal any further secretions or significant inflammation.

Considering the persistence of normal hematological and biochemical parameters, the patient underwent the removal of the antibiotic spacer and implantation of a megaprosthesis in February 2023. The surgical approach followed the deltopectoral approach utilized in previous procedures. Routine antibiotic prophylaxis involved treatment with teicoplanin. The periarticular tissue leukocyte count and rapid alpha-defensin test were performed again, and both results turned negative. Cultures of the antibiotic spacer and periarticular tissues showed negative results.

The glenoid component was positioned using a Global Positioning System (GPS) navigation system (Exactech, Inc., Gainesville, Florida, United States), and the humeral component was implanted using a modular stem. For the glenoid component, Equinoxe® Reverse Shoulder (Exactech, Inc.) with a 38 mm glenosphere fixed with two 30 mm compression screws was used. The humerus was replaced using Equinoxe® Humeral Reconstruction Prosthesis (Exactech, Inc.) with an extra-small proximal body (+0 mm), a cemented 11x80 mm stem, a distal stem collar of 20.5 mm, and a 38 mm constrained humeral liner.

Case 2

A 79-year-old female was evaluated and treated for severe functional limitation and major pain in right shoulder caused by a fracture resulting from the proximal humerus. The patient's medical history included arterial hypertension, dyslipidemia, and atrial fibrillation. She led an active life, completely autonomously. In November 2022, she underwent in another clinic a surgical procedure of intramedullary nail fixation for comminuted proximal humeral fracture (AO/OTA 11C3).

**Figure 5:**
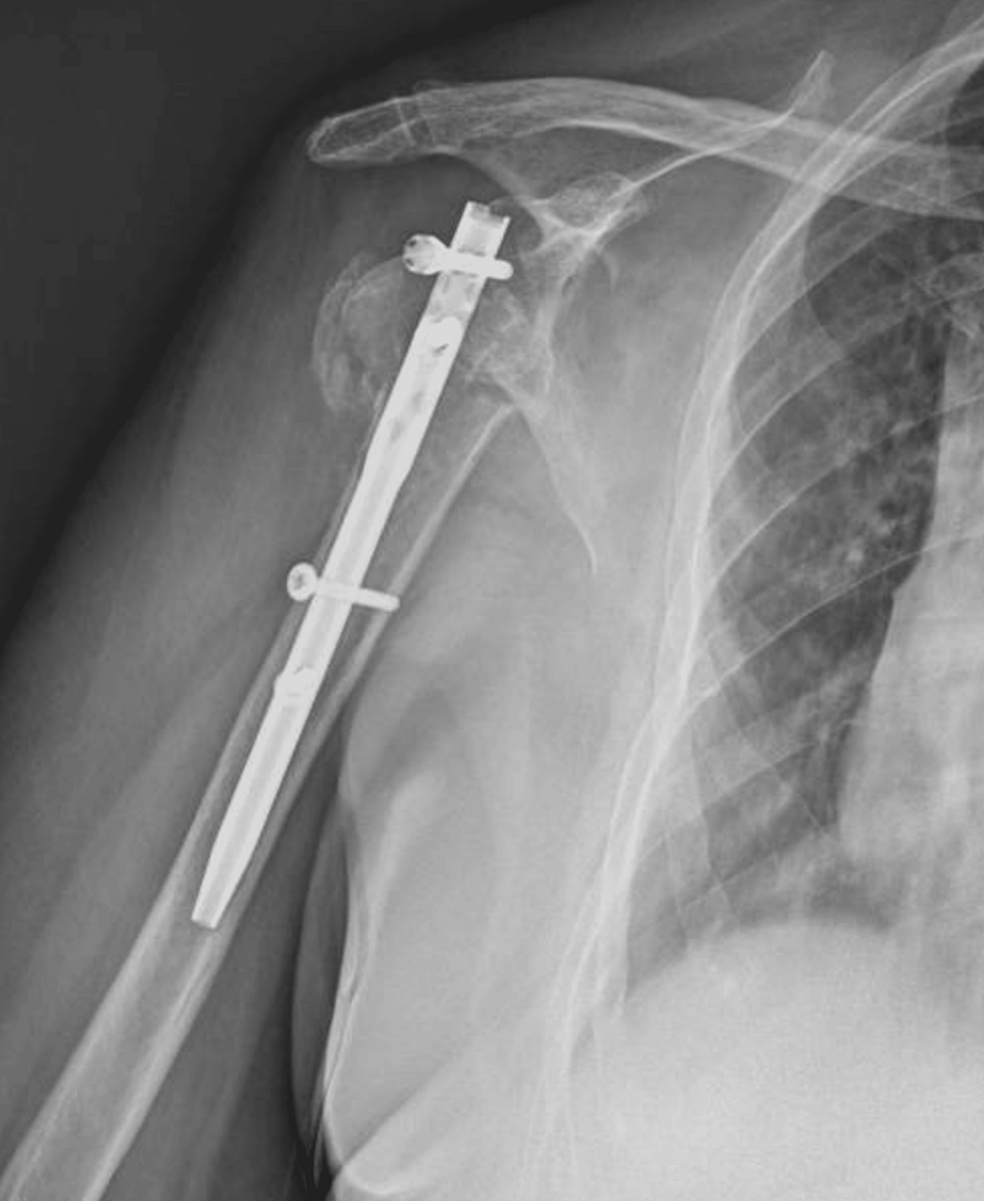
X-ray for evaluation of nail osteosynthesis failure (Case 2)

At our first evaluation in January 2023, in the orthopedic emergency room, the patient reported a disabling range of motion with abduction at 25°, passive elevation at 30°, abolition of rotational movements, and pain with visual analog scale (VAS) score of 8/10. An x-ray and a CT scan were performed which showed nail cutout resulting in acromial impingement, fracture valgus displacement, and severe bone loss. (Figure 5). 

After evaluating the clinical examination, imaging, and blood tests with inflammatory markers, the surgical indication was determined by complete joint impotence, degree of bone stock loss, and low functional demand in daily life.

A one-stage operation was performed in February 2023. The first step involved the removal of the fixation devices (intramedullary nail and fixing screws) and the second step involved the implantation of reverse shoulder arthroplasty. The glenoid component was implanted using a GPS navigation system (Exactech, Inc.), while the humeral component was performed by a use of a modular stem.

The patient received routine perioperative prophylactic antibiotic therapy with cefazolin. The surgery was performed using the deltopectoral approach. Fixation devices were removed and sent for culture (negative result). A leukocyte count was performed on the periarticular tissue, and a value less than of the cut-off of 5 granulocytes/5HPF was found.

For the glenoid component, Equinoxe Reverse Shoulder with a 38 mm glenosphere and a superior augment of 10° was used, fixed with three compression screws (two of 30 mm and one of 26 mm). The humerus replacement was performed using Equinoxe Humeral Reconstruction Prosthesis with a proximal body of medium size (+12.5 mm), a cemented 11x80 mm stem, a distal stem collar of 21.5 mm, and a 38 mm constrained humeral liner.

Rehabilitation

Postoperative rehabilitation protocols included immediate passive mobilization up to 90° in abduction and elevation, isometric deltoid exercises, full elbow flexion and extension, and no rotation. An adduction sling was used for the first 30 days. Rotational movements were gradually introduced after one month from the surgery.

Follow-up

**Figure 6:**
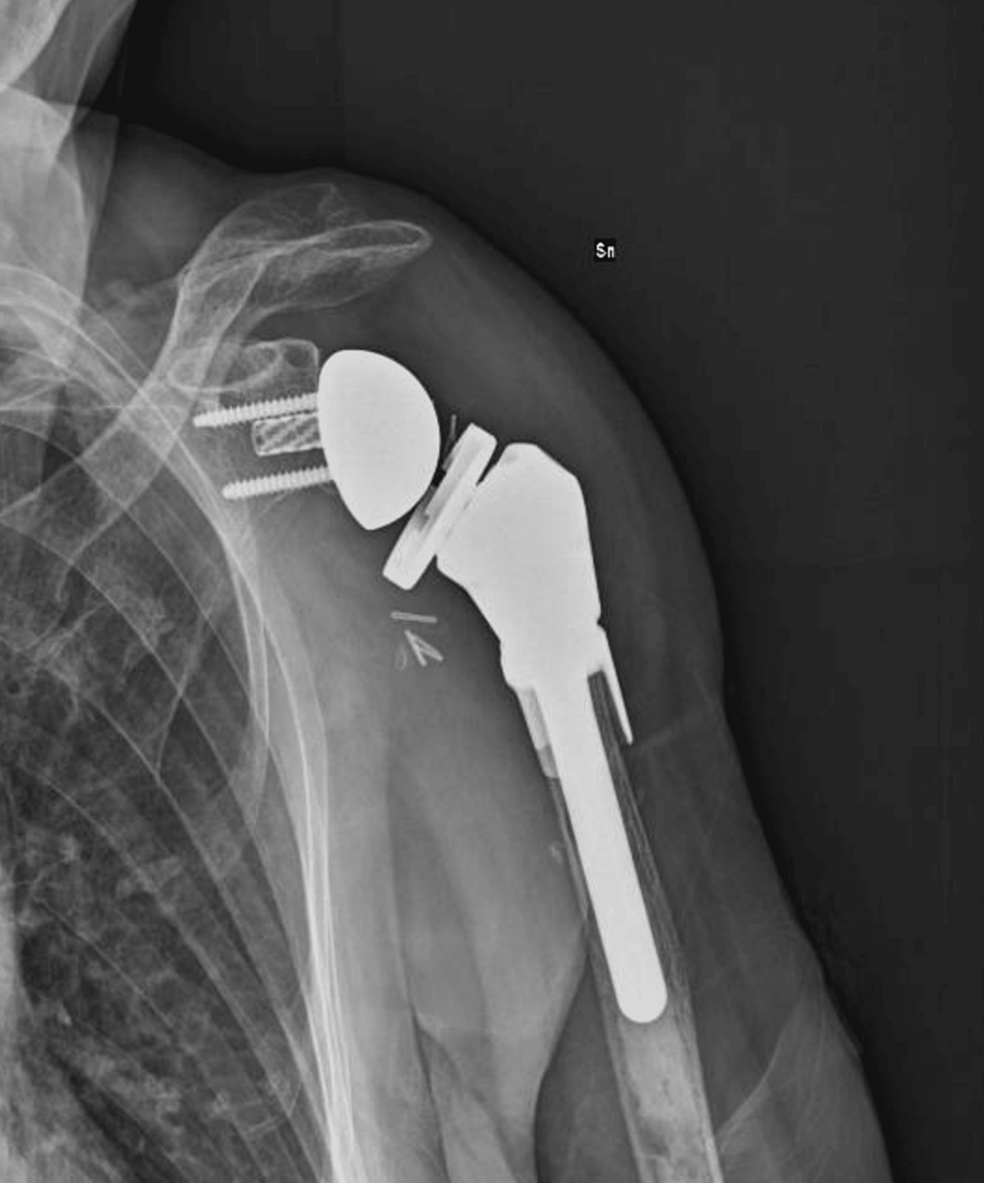
Twelve-month post-megaprosthesis X-ray evaluation (Case 1) Sn: left

**Figure 7:**
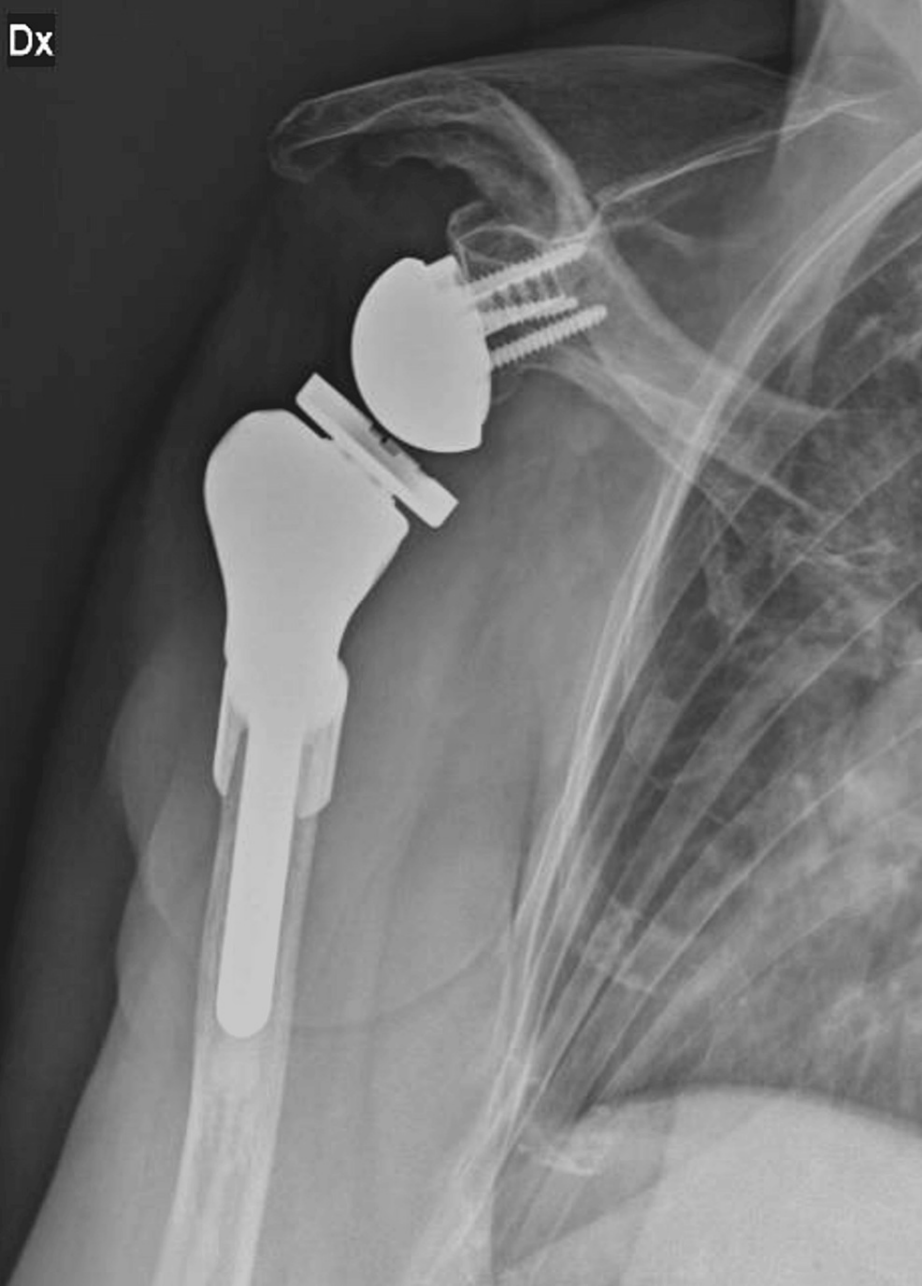
Twelve-month post-megaprosthesis X-ray evaluation (Case 2) Dx: right

After the surgical procedure, the patients followed our shoulder arthroplasty protocol: clinical control at one, three, six, and 12 months. Radiographic follow-up was performed at three, six, and 12 months (Figure 6 and Figure 7).

Currently, the patients show a satisfying functional recovery and increased quality of life. The different outcomes including the American Shoulder and Elbow Surgeons (ASES) Shoulder Score and Simple Shoulder Test (SST) before surgery and at different follow-up times after the replacement procedure are reported for both patients in Table 1.

**Table 1:** Outcomes data before megaprosthesis surgery and at different follow-ups ASES: American Shoulder and Elbow Surgeons; VAS: visual analog scale; SST: Simple Shoulder Test

	Time	Case 1	Case 2
Strength (5-point scale)	Before surgery	2	2
	At 3 months	2	3
	At 12 months	4	4
VAS (pain)	Before surgery	6	7
	At 3 months	1	1
	At 12 months	0	0
Abduction (deg)	Before surgery	25°	20°
	At 1 month	50° (passive)	75° (passive)
	At 12 months	60°	80°
Elevation (deg)	Before surgery	30°	30°
	At 1 month	50° (passive)	70° (passive)
	At 12 months	60°	80°
External rotation (deg)	Before	15°	20°
	At 1 month	20° (passive)	25° (passive)
	At 12 months	30°	35°
ASES Shoulder Score	Before	19	23
	At 12 months	57	65
SST	Before surgery	21	27
	At 12 months	61	69

**Figure 8:**
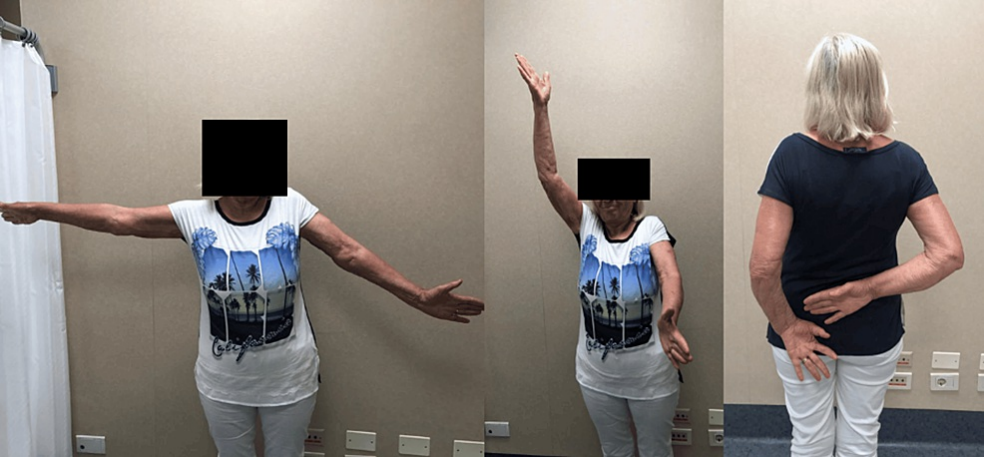
Range of motion at the 12-month postoperative clinical evaluation (Case 1)

**Figure 9:**
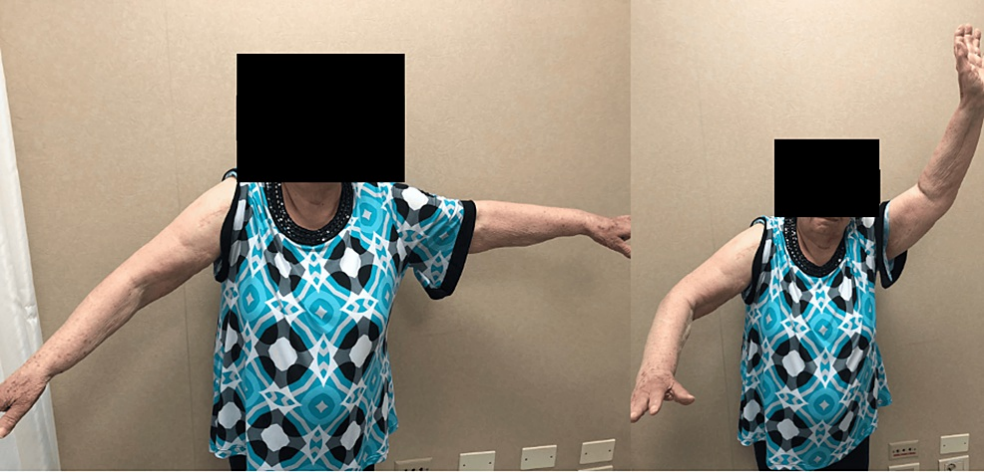
Range of motion at the 12-month postoperative clinical evaluation (Case 2)

Range of motion data was collected at the three-month follow-up after the surgery for passive movements and at the 12-month follow-up for active movements (Figure 8 and Figure 9). 

The next clinical and radiographic monitoring are scheduled at two years.

## Discussion

Resecting the bone tumor with oncologic safe margins is the primary objective of orthopedic oncologic surgery. Wide resections involve the excision of the tumor along with a cuff of surrounding, healthy, normal tissue around it. As a result, it becomes difficult to achieve an oncologically safe resection and preserve as much functional ability as feasible without generating an unstable joint. In recent years, the possibilities for reconstruction have grown, and, with improved surgical options, postoperative functional abilities have increased. Anatomical hemiarthroplasty, allograft-prosthesis composite, and reverse shoulder megaprosthesis are now popular surgical alternatives. As a result, this prosthesis is also appropriate for shoulder joint repair following oncologic proximal humerus excision [4].

On the other hand, considering proximal humerus nonunions, especially with severe bone loss, a variety of therapeutic options are available, including open reduction and internal fixation with several fixation constructions, hemiarthroplasty, anatomic total shoulder arthroplasty, and reverse shoulder arthroplasty [6]. While there is a consensus on the use of megaprosthesis in oncologic shoulder surgery [4], a similar consensus does not yet exist for proximal humerus nonunions.

The above contrasts with what can be observed in trauma surgery of the lower limb. Megaprosthesis is frequently used in lower limb surgery, not only in oncologic surgery but also in the treatment of trauma outcomes [7].

Nowadays, considering the advancing biological age of the general population and the implications of orthopedic surgery on the quality of life, it is necessary to personalize the treatment, considering the patient's characteristics and the condition of the surgical site. Moreover, based on the assumption that the functional outcomes of tumor megaprosthesis in shoulder surgery are associated with good medium- and long-term functional outcomes, we aimed to consider such treatment in the management of the case reports described above.

The cases in this report showed improvement in active and passive mobilization of the shoulder. Functional scores submitted to patients showed an increase in absolute values between preoperative and postoperative outcomes. Looking at Table 1, an improvement in functional scores during follow-up was observed. In addition, we recorded an improvement in strength assessed with the 5-point scale and also a reduction in subjective pain.

When assessing the range of motion, in particular at the three-month follow-up, we considered the passive range of motion. This is conditioned by the fact that during the first month our rehabilitation protocol involves only passive mobilization assisted by physical therapists. Moreover, regarding external rotation, we recorded small improvements in contrast to what we observed in both abduction and anterior elevation. The above is in line with the international literature referring to oncologic shoulder surgery [4,6].

At the 12-month follow-up, no complications have occurred in the two cases to date. Regarding the risk of complications, especially the risks of mobilization of the megaprosthesis, the CT-based intraoperative navigation system optimizes the configuration of the screw for the initial fixation of the glenoid component. Literature reports that primary fixation and reduction of micromotion offer an ideal biologic environment for osseointegration, particularly in older patients who are typically osteoporotic. This is critical to the success of glenoid baseplate fixation in reverse shoulder arthroplasty. Screw purchase length has been shown to have a significant impact on baseplate fixation and micromotion in experimental models of reverse shoulder arthroplasty. In order to achieve primary fixation of the glenoid component during reverse shoulder arthroplasty and preserve bone stock, intraoperative navigation enhances baseplate screw placement, allowing for a better screw configuration [8].

Three different databases (PubMed, Scopus, and Google Scholar) were searched for relevant articles, and further references were obtained by cross-referencing. Seventy-six studies met the inclusion criteria, reporting on cases of megaprosthesis in non-oncologic scenarios, but none of them specifically addressed the proximal humerus. Only two cases of proximal humerus megaprosthesis were reported in a non-oncologic setting in the literature. The two case reports present encouraging short-term data, both in terms of functional recovery and the absence of short-term complications [9,10].

It should be taken into consideration that megaprosthesis can serve as a viable option for orthopedic surgeons in selected extreme cases with severe bone loss. Patients with severe bone loss should be treated differently from oncology patients due to their longer life expectancy. Consequently, the surgical technique and implantation system must be meticulously executed to ensure the longevity of the prosthesis. The unique bone and soft tissue conditions in these patients pose critical challenges that surgeons must adeptly manage to avoid serious complications [11].

## Conclusions

The use of a humeral reconstruction prosthesis during reverse total shoulder arthroplasty is emerging as a promising solution, extending its application beyond the confines of oncologic interventions to encompass instances of unsuccessful trauma surgeries as well. This approach demonstrates significant potential in enhancing patient outcomes, particularly in terms of functionality. When evaluating the efficacy of this surgical technique, both functional scores and the range of motion serve as critical indicators.

The preliminary results are indeed promising, showcasing an improvement in the patients' quality of life which cannot be understated. However, it is crucial to acknowledge that the current body of evidence, while optimistic, is still in its nascent stages. There is a pressing need for more comprehensive studies, accompanied by extended periods of follow-up. Such research endeavors are essential to fully understand and delineate the capabilities and long-term viability of humeral reconstruction prostheses in the realm of trauma surgery.
